# Vitamin D epimers are associated with circulating haemoglobin levels independently of C-reactive protein

**DOI:** 10.1038/s41598-021-00086-z

**Published:** 2021-10-20

**Authors:** La-or Chailurkit, Piyamitr Sritara, Prin Vathesatogkit, Sukit Yamwong, Nisakron Thongmung, Boonsong Ongphiphadhanakul

**Affiliations:** 1 Division of Endocrinology and Metabolism, Department of Medicine, Faculty of Medicine, Ramathibodi Hospital, Mahidol University, Rama 6th Road, Bangkok, 10400 Thailand; 2 Research Center, Faculty of Medicine, Ramathibodi Hospital, Mahidol University, Bangkok, Thailand

**Keywords:** Endocrinology, Medical research

## Abstract

Vitamin D deficiency has been shown to be associated with anaemia. Circulating 25(OH)D consists of both epimeric and nonepimeric forms. However, the relative roles of epimeric and nonepimeric vitamin D in regulating anaemia and haemoglobin levels remain unknown. Therefore, in this study, we examined the effect of vitamin D, including its epimers, on haemoglobin levels, independently of its effect on circulating high-sensitivity C-reactive protein (hsCRP). This was a cross-sectional study of 1655 subjects from a long-term follow-up cohort at the Electricity Generating Authority of Thailand. Venous blood sample were collected for determination of vitamin D [25(OH)D_2_, 25(OH)D_3_, 3′-epi-25(OH)D_2_, and 3′-epi-25(OH)D_3_], haemoglobin, and hsCRP levels. Data are presented as mean ± standard deviation. Age, sex, and body mass index (BMI) were significantly associated with circulating haemoglobin levels, while no association was found between total serum 25(OH)D and haemoglobin levels. However, when total 25(OH)D was separated into 3′-epimeric and non-3′-epimeric forms, 3′-epi-25(OH)D was significantly associated with haemoglobin levels, independently of age, sex, and BMI (*P* < 0.01). No association was found between non-3′-epi-25(OH)D and haemoglobin. When hsCRP was added to the model, the effect 3′-epi-25(OH)D on haemoglobin levels remained significant (*P* < 0.01). In conclusion, vitamin D epimers are associated with circulating haemoglobin levels, which supports the role of vitamin D in red blood cell and iron physiology.

## Introduction

Vitamin D plays essential roles in many physiological processes in addition to calcium and bone metabolism^1^, and vitamin D receptors are expressed in numerous tissues throughout the body. In circulation, vitamin D exists in epimeric and nonepimeric forms^2^. Although the relative proportions of these forms may influence biochemical assays and interpretation of vitamin D status^3^, the similarities and distinctions in their biological functions are less clear. However, differences in the degree and direction of the association between the epimeric and nonepimeric forms of vitamin D and serum lipid levels^4^ and incident diabetes^5^ have been demonstrated.

Regarding erythropoiesis and anaemia, previous studies showed it may be improved in response to vitamin D administration^6–8^, which may be mediated by decreasing proinflammatory cytokines and hepcidin, resulting in increased iron availability^9^. Moreover, administration of high-dose vitamin D in intensive care unit patients was shown to result in improvement of anaemia^10^. Vitamin D exerts diverse immunomodulatory functions, and vitamin D receptor activation can reduce proinflammatory cytokines^11^. However, it remains unclear if the mechanism underlying the effect of vitamin D on anaemia is mediated in part by the suppressive effect of vitamin D on inflammation. Moreover, it is currently unknown how the different epimeric forms of vitamin D affect erythropoiesis. Therefore, in the present study, we conducted a cross-sectional study of 1655 subjects at the Electricity Generating Authority of Thailand (EGAT) to investigate the effect of vitamin D, including its epimers, on haemoglobin levels, independent of its effect on circulating C-reactive protein.

## Methods

### Study population

Participants (n = 1655) were recruited from a subsample of subjects from the population studied in the EGAT study in 2009 (EGAT 3). Details on the study cohort were reported previously^12^. Briefly, subjects in the cohort were employees of EGAT who volunteered to participate in a health survey. The exclusion criteria were acute or chronic conditions including advanced cancer, incapacitating neurological or cardiovascular disease and psychiatric illnesses which would limit the ability of the subject to participate in the study or refusal of give informed consent.All participants completed a medical evaluation and underwent routine laboratory investigations, including urinalysis. Blood was drawn after a 12-h fast. In summary, there were a total of three EGAT cohorts. In 1985, 3499 EGAT employees (half of total employees) were randomly enrolled as the EGAT 1 cohort. In 1998, 2999 employees were randomly enrolled as the EGAT 2 cohort. Finally, in 2009, 2584 participants were recruited as the EGAT 3 cohort. EGAT 3 was resurveyed in 2014. Each time, the same individuals were contacted by telephone and invitation letter to attend the follow-up examination. Otherwise, information on cause of death was sought for those known to have died during the interim period. At each follow-up visit, subjects underwent similar medical evaluations and identical routine laboratory investigations as during the baseline visit. Fasting blood samples were obtained. A portion of blood samples was collected in anticoagulant blood tubes (EDTA-K2) for immediate routine blood tests, while the remainder was transferred to blood collection tubes (without additives) for separation of serum. Serum was separated by centrifugation and stored at − 80 °C for future analysis. The Committee on Human Rights Related to Research Involving Human Subjects, Faculty of Medicine, Ramathibodi Hospital, Mahidol University, approved the study, and it conformed to the provisions of the Declaration of Helsinki (as revised in Fortaleza, Brazil, October 2013). All participants provided written informed consent before participating in the study.

### Measurement of serum vitamin D levels (as previously described^13^)

Serum levels of 25(OH)D_2_, 25(OH)D_3_, 3′-epi-25(OH)D_2_, and 3′-epi-25(OH)D_3_ were analysed using liquid chromatography-tandem mass spectrometry. An Agilent 1260 Infinity Liquid Chromatograph (Agilent Technologies, Waldbronn, Germany) was used, coupled to a QTRAP^®^ 5500 tandem mass spectrometer (AB SCIEX, Framingham, MA, USA) using a MassChrom^®^ 25-OH-D3/D2 serum/plasma diagnostics kit with a 3′-epi-25-OH-D3/D2 upgrade (Chromsystems, Munich, Germany). An atmospheric pressure chemical ionization source, operated in positive mode, was used to ionize the target compound. Data were collected and analysed using Analyst Software, Version 1.6.2 (Applied Biosystems, USA). All calibrators (Chromsystems 3PLUS1^®^ Multilevel Serum Calibrator Set 3′-epi-25-OH-D3/D2 and 25-OH-D3/D2) and serum controls (MassCheck^®^ 3′-epi-25-OH-D3/D2 and 25-OH-D3/D2) used in this study were traceable to certified substances and standard reference materials (SRM) of the National Institute of Standards and Technology (NIST; SRM 972, Gaithersburg, MD, USA). Each batch contained a four-point calibration curve and two levels of quality control materials. Acceptable linearity of calibration curves was achieved when correlation coefficients were 0.998 or higher. In this study, total serum 25(OH)D comprised the sum of 25(OH)D_3_, 25(OH)D_2_, 3′-epi-25(OH)D_2_, and 3′-epi-25(OH)D_3_. Separated by C3′-epimeric form, non-C3′-epimers were the sum of 25(OH)D_2_ and 25(OH)D_3_, and C3′-epimers were the sum of 3′-epi-25(OH)D_2_ and 3′-epi-25(OH)D_3_. The coefficients of variation of 25(OH)D_3_, 25(OH)D_2_, and 3′-epi-25(OH)D_3_ were 9.2, 19.9, and 11.8, respectively.

### Measurement of haemoglobin

Haemoglobin concentration was measured using an automated haematology analyser (Mindray BC-6800Plus, Mindray Bio‐Medical Electronics Co., Ltd, Shenzhen, China).

### Measurement of serum high-sensitivity C-reactive protein

Serum high-sensitivity C-reactive protein *(*hsCRP) was measured using particle-enhanced immunonephelometry with CardioPhase^®^ hsCRP on a BN ProSpec System (Siemens Healthcare Diagnostics, Liederbach, Germany). Both the lower limit of detection and lower limit of quantification were equal to 0.175 mg/L according to the manufacturer's instructions.

### Statistical analyses (as previously described^4^)

Normally distributed data are presented as mean ± standard deviation and non-normally distributed data are presented as median and range. The relative 3′-epimer contribution (%) was used to express the amount of 3′-epimer-25(OH)D as a percentage of total 25(OH)D (the sum of 25(OH)D and 3′-epi-25(OH)D). The Kolmogorov–Smirnov test was used to test for normality. Logarithmic transformation was performed when data did not follow a normal distribution. The Mann–Whitney U test was used for comparisons of differences between two independent groups. Correlations between two parameters were estimated using Spearman's rank correlation coefficient. Multiple linear regression models were used to assess potential associated factors. *P* < 0.05 was considered statistically significant. All analyses were performed using Stata Statistical Software, Release 12 (StataCorp, College Station, TX, USA).

## Results

Clinical features of the study population are shown in Table 1. Most subjects were male because of the demographic structure of the employees of EGAT. Mean age was 40 ± 7 years, body mass index (BMI) was 24 ± 4, and circulating total 25(OH)D was 25 ± 7 ng/mL Overall, 365 (22%) subjects had vitamin D deficiency defined as 25(OH)D < 20 ng/mL, and 987 (60%) had vitamin D insufficiency defined as 25(OH)D ≥ 20 ng/mL and < 30 ng/mL and hsCRP levels were 2.0 ± 3.6 mg/L.

**Table 1:** Clinical features of the study population.

Variable	Mean ± SD
Age (years)	40 ± 7
Male sex (%)	73
BMI (kg/m^2^)	24 ± 4
Haemoglobin (g/dL)	14 ± 2
High-sensitivity C-reactive protein (mg/L)	2.0 ± 3.6
Total 25(OH)D (ng/mL)	25 ± 7

The epimeric and non-epimeric forms of 25(OH)D_3_ and 25(OH)D_2_ and their contributions to total 25(OH)D levels according to 25(OH)D tertiles are shown in Table 2. The 3′-epimer of 25(OH)D_3_ comprised 6.2% of total 25(OH)D_3_ while the contribution of the 3′-epimer of 25(OH)D_2_ to total 25(OH)D_2_ was much lower at 0.3%.

**Table 2:** Composition of 25(OH)D according to its epimeric and nonepimeric forms according to 25(OH)D tertiles.

Variable	Total (ng/mL)	3′-epimer (ng/mL)	Non-3′-epimer (ng/mL)
**Total 25(OH)D**_**3**_** (ng/mL)**			
Low tertile	23 ± 6	1.5 ± 1.6	22 ± 5
Mid tertile	25 ± 6	1.5 ± 1.6	23 ± 6
High tertile	26 ± 7	1.6 ± 1.8	24 ± 7
Total	24 ± 7	1.5 ± 1.7	23 ± 6
**Total 25(OH)D**_**2**_** (ng/mL)**			
Low tertile	0.31 ± 0.09	0	0.31 ± 0.09
Mid tertile	0.50 ± 0.05	0	0.50 ± 0.05
High tertile	1.1 ± 3.5	0.01 ± 0.05	1.1 ± 3.5
Total	0.6 ± 2.1	0.002 ± 0.03	0.6 ± 2.1

**Figure 1:**
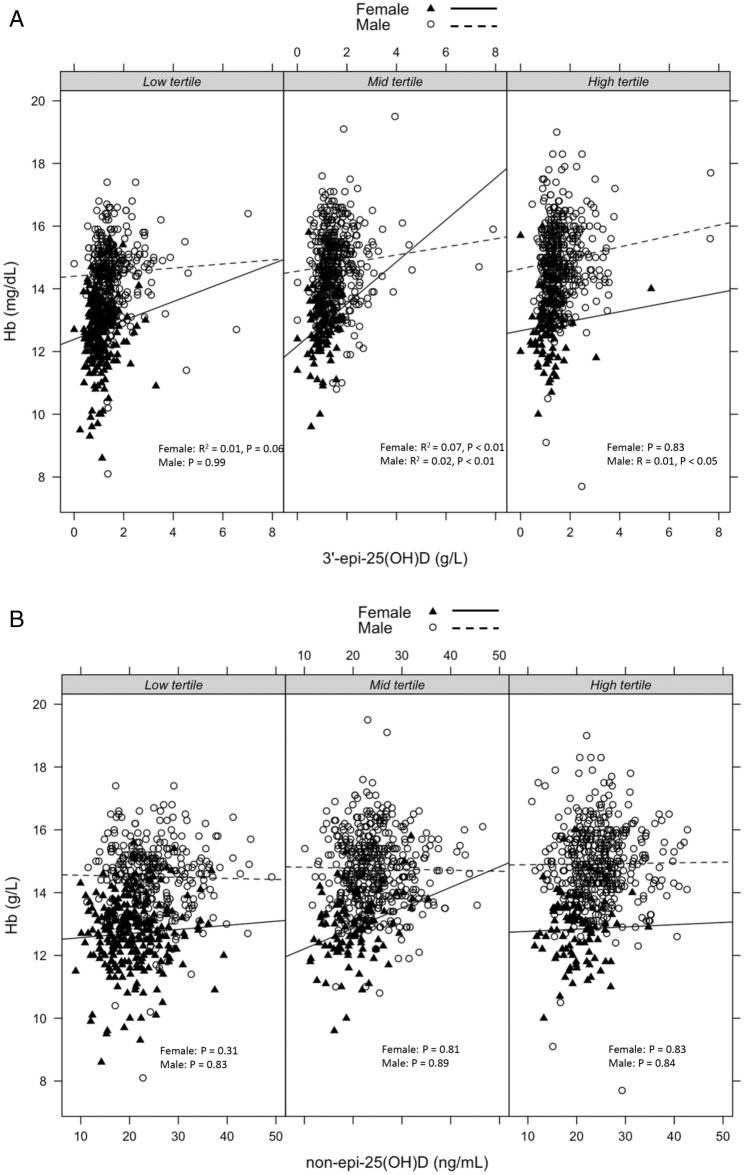
(**A**) Relationship between 3’-epi-25(OH)D and Hb levels according sex and BMI tertiles. 3’-epi-25(OH)D levels in either males or females were significantly related to Hb levels in all BMI tertiles. (**B**) Relationship between Hb and non-3’-epi-25(OH)D levels according sex and BMI tertiles. There was no significant relationship between non-epi-25(OH)D levels and Hb levels.

Using multiple linear regression to investigate variables associated with circulating haemoglobin levels, it was found that age, sex, and BMI were significantly associated with haemoglobin levels (beta − 0.05, *P* < 0.01; beta − 0.58, *P* < 0.001; and beta 0.09, *P* < 0.001, respectively). In contrast, total serum 25(OH)D was not significantly associated with haemoglobin levels (beta 0.02, *P* = 0.46). However, when total 25(OH)D was separated into epimeric and nonepimeric forms, 3′-epi-25(OH)D was significantly associated with haemoglobin levels in males or females according to BMI tertiles (Fig. 1A) while no such association was found with non-3’-epi-25(OH)D (Fig. 1B). 3′-epi-25(OH)D was significantly associated with haemoglobin levels (beta 0.05, *P* < 0.01), independently of age, sex, and BMI (Table 3). The effect of 3′-epi-25(OH)D was comparable with that of age, but in the opposite direction, as suggested by the beta coefficients. In contrast, no association was found between nonepimeric 25(OH)D and haemoglobin levels was found (beta 0.02, *P* < 0.46) (Table 4).

**Table 3:** Association between total 3′-epi-25(OH)D and haemoglobin levels.

Variable	Regression Coefficient ± SE	Beta	*P* value
Age (years)	− 0.01 ± 0.004	− 0.05	< 0.01
Female sex	− 0.9 ± + 0.07	− 0.58	< 0.001
BMI (kg/m^2^)	0.03 ± 0.01	0.08	< 0.001
Total 3′-epi-25(OH)D (ng/mL)	0.05 ± 0.02	0.05	< 0.01

Total 3′-epi-25(OH)D was associated with haemoglobin levels, independently of age, sex, and BMI.

**Table 4:** Lack of association between total non-3′-epi-25(OH)D and haemoglobin levels.

Variable	Regression Coefficient ± SE	Beta	*P* value
Age (years)	− 0.01 ± 0.004	− 0.05	< 0.01
Female sex	− 0.9 ± 0.07	− 0.58	< 0.001
BMI (kg/m^2^)	0.03 ± 0.01	0.09	< 0.001
Total non-3′-epi-25(OH)D (ng/mL)	0.004 ± 0.005	0.02	0.46

Total non-3′-epi-25(OH)D was not associated with haemoglobin levels after controlling for age, sex, and BMI.

Regarding systemic inflammation as assessed by serum hsCRP, higher levels of circulating hsCRP were associated with lower haemoglobin concentrations (beta − 0.07, *P* < 0.001), independently of age, sex, and BMI (Table 5). According to a regression model that also included 3′-epi-25(OH)D, the effect of 3′-epi-25(OH)D remained significant after controlling for hsCRP, age, sex, and BMI (beta 0.05, *P* < 0.01). The beta coefficients in the regression model suggested the effect of 3′-epi-25(OH)D was comparable with that of hsCRP, but in the opposite direction (Table 6). No association was identified between non-3′-epi-25(OH)D and haemoglobin in the presence of hsCRP, age, sex, and BMI (beta 0.02, *P* = 0.34) (Table 7).

**Table 5:** Association between hsCRP and haemoglobin levels. hsCRP was associated with haemoglobin levels, independently of age, sex, and BMI.

Variable	Regression Coefficient ± SE	Beta	*P* value
Age (years)	− 0.01 ± 0.004	− 0.05	< 0.05
Female sex	− 1.9 ± 0.07	− 0.58	< 0.001
BMI (kg/m^2^)	0.04 ± 0.01	0.11	< 0.001
Ln(CRP)	− 0.10 ± 0.03	− 0.07	< 0.001

**Table 6:** The association between total 3′-epi-25(OH)D and haemoglobin levels was independent of hsCRP.

Variable	Regression Coefficient ± SE	Beta	*P* value
Age (years)	− 0.01 ± 0.004	− 0.05	< 0.01
Female sex	− 1.9 ± 0.07	− 0.58	< 0.001
BMI (kg/m^2^)	0.04 ± 0.01	0.11	< 0.001
Total 3′-epi-25(OH)D (ng/mL)	0.05 ± 0.02	0.05	< 0.01
Ln(CRP)	− 0.10 ± 0.03	− 0.07	< 0.01

After controlling for hsCRP, total 3′-epi-25(OH)D remained significantly associated with haemoglobin levels, independently of other factors.

**Table 7:** Lack of association between total non-3′-epi-25(OH)D and haemoglobin levels, independent of hsCRP and other factors.

Variable	Regression Coefficient ± SE	Beta	*P* value
Age (years)	− 0.01 ± 0.004	− 0.05	< 0.05
Female sex	− 1.9 ± 0.07	− 0.58	< 0.001
BMI (kg/m^2^)	0.05 ± 0.01	0.11	< 0.001
Total non-3′-epi-25(OH)D (ng/mL)	0.005 ± 0.005	0.02	0.34
Ln(CRP)	− 0.10 ± 0.03	− 0.06	< 0.001

## Discussion

According to our results, systemic inflammation, as partly reflected by circulating hsCRP, was strongly associated with haemoglobin concentration. Systemic inflammation is well established as a factor that influences erythropoiesis, and can lead to anaemia of inflammation^14^. Pathogenic features of anaemia of inflammation include shortening of red cell lifespan, impaired erythropoietin production, blunted responsiveness of marrow to erythropoietin, and impaired iron metabolism mediated by inflammatory cytokines and the iron regulatory peptide, hepcidin^14,15^. Regarding the association between CRP and haemoglobin levels, in dialysis patients, high CRP levels were reported to be associated with low haemoglobin levels^16^. Similarly, a strong correlation between CRP and haemoglobin levels was demonstrated in children^17^. Our findings suggest further that mild systemic inflammation in relatively healthy adults is related to and play a role in determining haemoglobin levels.

Unlike with hsCRP levels, we found that total 25(OH)D levels did not correlate with haemoglobin levels. However, when total 25(OH)OD was stratified according to 3′ epimerization, the 3′-epimer was associated with haemoglobin levels, while the nonepimeric form was not. To our knowledge, there are no previous reports on the association between 3′-epi-25(OH)D and haemoglobin levels. There are increasing reports on differences in biological activity between the epimeric and nonepimeric forms of vitamin D metabolites. In addition to its discrepant effect on haemoglobin, 3′-epi-25(OH)D has been shown to have reduced binding to the vitamin D receptor, and lower calcaemic activity^18^. Moreover, a clinical study revealed there are discrepant associations between 25(OH)D and its epimers and serum lipid levels^4^ and incident diabetes^5^. Taken together, when assessing the biological activity of vitamin D, it is prudent to separately assess the roles of its epimeric and nonepimeric forms.

Numerous intervention studies have investigated the effect of vitamin D supplementation on haemoglobin levels, with contradictory results. In critically ill adults on mechanical ventilation, treatment with 500,000 IU D3, in 11 subjects as compared to 10 subjects taking placebo, increased haemoglobin levels and decreased hepcidin levels compared to the placebo group (n = 10)^7^. However, daily supplementation with 2800 IU vitamin D for 8 weeks, 100 subjects in the vitamin D group as compared to 100 subjects in the placebo group, did not improve haemoglobin levels in patients with hypertension^19^. According to a systematic review of 14 randomized clinical trials, vitamin D supplementation has no effect on haemoglobin or ferritin levels, whereas it has positive effects on transferrin saturation and iron status^7^. It should be noted, however, that vitamin D epimers were not assessed in these studies. It is therefore unclear how increased 3′-epi-25(OH)D is associated with improvement of anaemia status. Oral supplementation with vitamin D increases both the epimeric and nonepimeric forms of vitamin D. In mice, the degree of epimerization is greater with oral vitamin D supplementation than with exposure to ultraviolet radiation, while the same observation was not made in humans^20^. Further clinical trials that investigate the role of vitamin D in determining anaemia and iron status should examine the degree of epimerization as a determinant of responsiveness to vitamin D supplementation.

In terms of the underlying mechanisms for the association between the vitamin D epimer and hemoglobin levels, there has been evidence supporting the causal role vitamin D in erythropoiesis. For example, high-dose vitamin D3 significantly decreases circulating hepcidin, an inhibitor of gut enterocytes iron transport, without a change in pro-inflammatory cytokines levels^9^. With regard to the effect of vitamin D epimers in relation to the total or non-epimeric forms of 25(OH), it has been shown that the epimeric forms of vitamin D are more related to a number of disease states including type 2 diabetes, rheumatoid arthritis and Alzheimer’s disease^21^ as well as dyslipidemia^4^. This may be due to the difference in the activation of the vitamin D receptors of different structural forms of vitamin D^22^.

There is a number of limitations in the present study. The study was cross-sectional in nature and the causative role of vitamin D epimers, if any, could not be readily determined. Moreover, the number of variables examined was limited as data regarding concurrent medications or supplements and others which may influence hemoglobin levels were not available. Further studies taken such variables into account are therefore warranted.

## Conclusion

Vitamin D epimers are associated with serum haemoglobin. This observation supports the role of vitamin D in red blood cell and possibly iron physiology.

## Data Availability

The datasets generated and/or analysed during the present study are available from the corresponding author upon reasonable request.
